# The role of Th17 cells/IL-17A in AD, PD, ALS and the strategic therapy targeting on IL-17A

**DOI:** 10.1186/s12974-022-02446-6

**Published:** 2022-04-22

**Authors:** Jiajia Fu, Yan Huang, Ting Bao, Chengcheng Liu, Xi Liu, Xueping Chen

**Affiliations:** 1 Department of Neurology, West China Hospital, Sichuan University, Wai Nan Guo Xue Xiang 37#, Chengdu, Sichuan China; 2 Management Center, West China Hospital, Sichuan University, Chengdu, Sichuan China; 3 State Key Laboratory of Oral Diseases, National Clinical Research Center for Oral Diseases, Department of Periodontics, West China Hospital of Stomatology, Sichuan University, Chengdu, Sichuan China; 4 Department of Neurology, The Second Affiliated Hospital of Chongqing Medical University, Chongqing, China

**Keywords:** TH17, IL-17A, Alzheimer’s disease, Parkinson’s disease, Amyotrophic lateral sclerosis, Targeted therapy

## Abstract

Neurodegenerative diseases are a group of disorders characterized by progressive loss of certain populations of neurons, which eventually lead to dysfunction. These diseases include Alzheimer’s disease (AD), Parkinson’s disease (PD), and amyotrophic lateral sclerosis (ALS). Immune pathway dysregulation is one of the common features of neurodegeneration. Recently, there is growing interest in the specific role of T helper Th 17 cells and Interleukin-17A (IL-17A), the most important cytokine of Th 17 cells, in the pathogenesis of the central nervous system (CNS) of neurodegenerative diseases. In the present study, we summarized current knowledge about the function of Th17/IL-17A, the physiology of Th17/IL-17A in diseases, and the contribution of Th17/IL-17A in AD, PD, and ALS. We also update the findings on IL-17A-targeting drugs as potentially immunomodulatory therapeutic agents for neurodegenerative diseases. Although the specific mechanism of Th17/IL-17A in this group of diseases is still controversial, uncovering the molecular pathways of Th17/IL-17A in neurodegeneration allows the identification of suitable targets to modulate these cellular processes. Therapeutics targeting IL-17A might represent potentially novel anti-neurodegeneration drugs.

## Background

Neurodegenerative diseases are a group of disorders characterized by progressive loss of certain populations of neurons, which eventually lead to dysfunction. These diseases include Alzheimer’s disease (AD), Parkinson’s disease (PD), and amyotrophic lateral sclerosis (ALS). At present, the treatment of neurodegenerative diseases is still very difficult, so it is very important to understand the pathophysiological mechanism of neurodegenerative diseases. Neurodegenerative diseases are characterized by selective susceptibility of certain nerve cells, different protein aggregation, and abnormal immune responses [1]. The pathogenesis of neurodegeneration is the joint action of many factors, and neuroinflammation is considered to be part of the cause of neurodegeneration. Neuroinflammation is characterized by elevated levels of inflammatory mediators or cytokines in the central nervous system (CNS) parenchyma [2]. Recently, there is growing interest in the specific role of T helper 17 (TH17) cells and Interleukin-17A (IL-17A), the most important cytokine of Th 17 cells, in the pathogenesis of the CNS of neurodegenerative diseases. Studies have shown that IL-17A acts on multiple resident cells of the central nervous system, enhances neuroinflammatory response, and plays a pathogenic role in a variety of neurodegenerative diseases [3]. However, the role of TH17/IL-17A in neurodegenerative diseases is still unclear and contradictory. Therefore, we summarized current knowledge about the function of Th17/IL-17A, the physiology of Th17/IL-17A in diseases, and the contribution of Th17/IL-17A in AD, PD, and ALS. We also update the findings on IL-17A-targeting drugs as potentially immunomodulatory therapeutic agents for neurodegenerative diseases.

## Biology of Th17 cells and IL-17A

Th17 cells were recognized in 2005 as a distinct lineage of T helper (Th) CD4+ cells [4, 5]. The differentiation of Th17 cells requires stimulation with certain cytokines, including IL-6, IL-23, IL-1β, transforming growth factor-β (TGF-β), and IL-21 [6–14]. These cytokines can trigger the JAK–STAT3 axis, and increase the expression of transcription factors, including retinoic orphan receptor (ROR)γt and RORα [15–19]. Th17 cells would achieve the pathogenic potential under the stimulation by pro-inflammatory cytokines IL-6, IL-23, and IL-1β, whereas cytokine TGF-β drives the development of protective Th17 cells by inducing the production of anti-inflammatory cytokine IL-10 [19–21]. IL-21 stimulates the expansion of Th17 cells in an autocrine loop [22]. IL-17A, initially called cytotoxic T-lymphocyte antigen (CTLA)-8 and cloned firstly in 1993, is the signature cytokine of Th17 cells [23], and it was described as an RNA transcript homologous to a Herpesvirus Saimiri gene. In 1995, the IL-17-binding receptor was first reported [24, 25]. Besides Th17 cells, other variable sources also produce IL-17A, including γδT, T-cell receptor (TCR)-β+ natural Th17, natural killer T (NKT), group 3 innate lymphoid cells (ILC3), Paneth cells, macrophages, and microglia in the CNS [26–29].

## The function of Th17 cells and IL-17A

First, Th17 cells can trigger pro-inflammatory danger signals, recruit and activate neutrophil granulocytes, upregulate the expression of antimicrobial factors, and promote the clearance of extracellular bacteria and fungi [30, 31]. IL-17A has an important capacity to induce the expression of chemokines and cytokines [3]. The chemokines, including C-X-C motif ligand 1 (CXCL1), CXCL2, and CXCL8 can attract myeloid cells to infected or injured tissues [32]. The cytokines, including granulocyte colony-stimulating factor (G-CSF) and IL-6 can promote myeloid-driven innate inflammation [33]. The pro-inflammatory cytokines and antimicrobial peptides are upregulated to put a synergistic effect on limiting fungal overgrowth [34, 35]. For example, in healthy skin, the IL-17A production is induced by commensal microflora to provide anti-fungal protection [23]. When the epithelial barrier of the skin is destroyed by injury, IL-17A can promote the proliferation of epithelial cells and the clearance of the pathogenic agents [36]. In the intestine, the IL-17A production is driven by the microbiota from the local epithelium to provide the antimicrobial function, and it can be helpful to control dysbiosis and to maintain a homeostatic balance in the gut [37, 38]. In the lamina propria of the small intestine, Th17 cells can mediate the protection against pathogenic microorganisms. In the brain of AD patients, Malassezia species, one of the most common fungi detected can lead to neuroinflammation via activating Th17 immune response [39].

Second, Th17 cells and IL-17A are mainly pro-inflammatory, and they are considered to be associated with several autoimmune diseases, including psoriasis, ankylosing spondylitis (AS), rheumatoid arthritis (RA), systemic lupus erythematosus (SLE), and inflammatory bowel disease (IBD) [40]. In psoriasis, the pathogenic inflammation was promoted by dysregulated IL-17 signaling. Th17 cells could infiltrate the psoriatic skin lesions, and inhibition of IL-17A had an effective treatment for psoriasis [41]. In AS, Th17 cells and IL-17A contribute to pathogenic inflammation, and it is effective to use an anti-IL-17A monoclonal antibody to treat AS [42]. In patients with RA, IL-17A was present at the sites of inflammatory arthritis, and higher numbers of IL-17+ CD4+ T cells were found in peripheral blood, but the efficacy of brodalumab, a human anti-IL-17A monoclonal antibody, in the treatment of RA was negative [40, 43, 44]. In patients with SLE, increased levels of IL-23, IL-21, and IL-17 were identified, which was associated with the expansion of Th17 cells [40, 45]. In patients with IBD, high levels of IL-17 and IL-21 in serum were reported [40, 46].

Third, the role of Th17 cells and IL-17A as indicated in the pathogenesis of CNS autoimmune disorders. Multiple sclerosis (MS) is a chronic CNS inflammatory disease, and the most characteristic animal model of MS is experimental autoimmune encephalomyelitis (EAE), used to explore the pathogenesis of MS. Th17 cells are one of the key effectors in MS and EAE, and MS was marked as a primarily IL-17-mediated autoimmune disease [47]. In MS patients, the expression of IL-17A and Th17-associated transcript IL-6 was increased in the demyelinated plaques [48], and the gene expression of IL-17 ranked at the highest in the CNS at autopsy [48]. The IL-17 level in serum was higher in MS patients with relapses and remissions [49], with an association to disease activity [50]. The proportion of Th17 cells in serum was increased during relapses [51, 52]. In the cerebral fluid (CSF), IL-17A level was elevated in patients with relapses and remissions, with a correlation to the level of the blood–brain barrier (BBB) dysfunction [53]. The EAE mouse model showed that Th17 cells could infiltrate the brain [54] and IL-17 could disrupt BBB [55]. In the cell model, Th17 cells were proved to cross the BBB, and the presence of Th17 cells in the lesions of CNS was related to enhanced neuroinflammation [56]. Th17 cells contribute to the disruption of the BBB [57], promote the activation of astrocytes and microglia within the CNS, and amplify neuroinflammation in EAE by targeting resident glial cells [58, 59]. Studies have shown that IL-17 neutralization could attenuate EAE progression through alleviating the generation of pathogenic cytokines [60], and EAE severity could be ameliorated in IL-17-deficient mice [61–63]. Phase IIa study of secukinumab showed that an IL-17A-neutralizing monoclonal antibody might be effective in reducing MRI lesion activity in MS [64].

### Th17 cells and IL-17A in neurodegenerative diseases

**Fig. 1:**
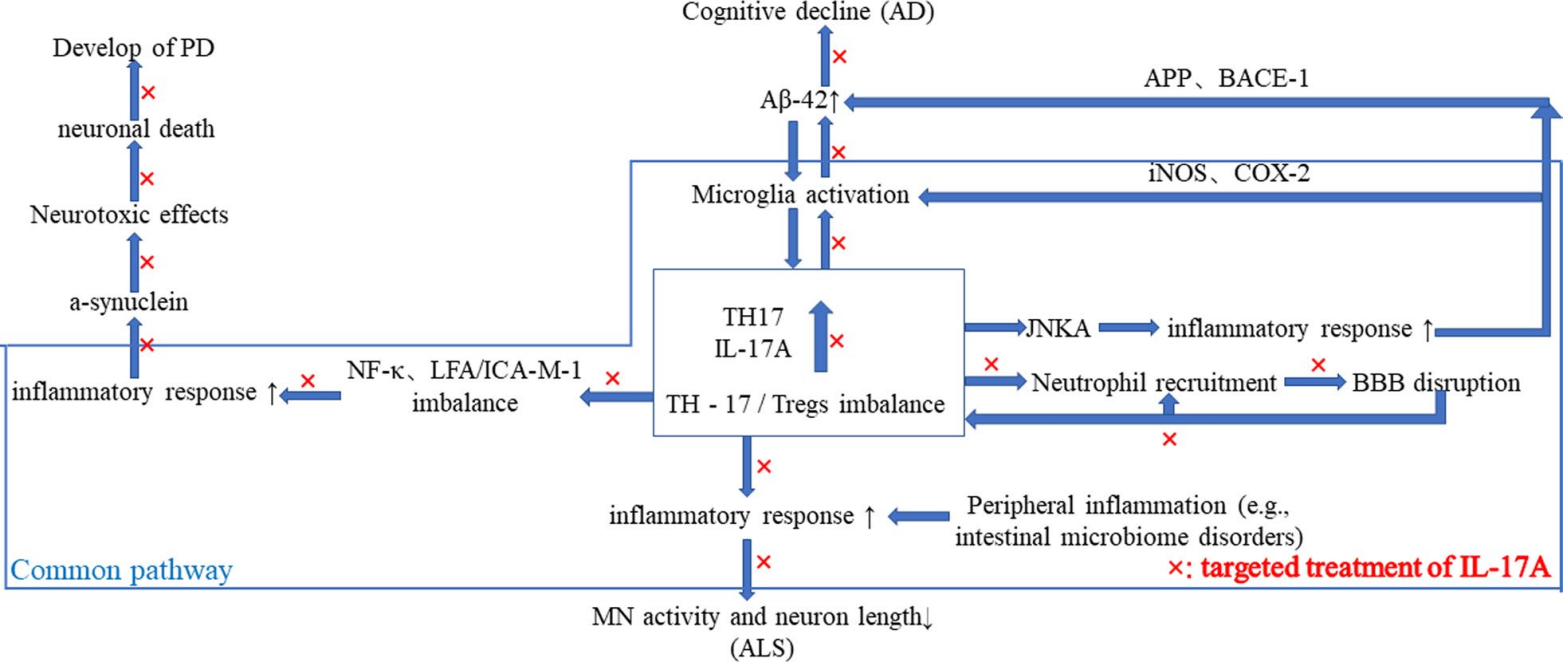
The function of TH17/IL17-A in AD, PD, and ALS

Neurodegenerative diseases are characterized by the selective vulnerability of certain neuronal cells, diverse protein aggregation, and abnormal immune responses [1]. Studies have shown that IL-17A played a pathogenic role in several neurodegenerative diseases [3]. Regarding the contribution of Th17 cells and IL-17A in Alzheimer’s disease (AD), Parkinson’s disease (PD), and amyotrophic lateral sclerosis (ALS), we systematically retrieved and critically evaluated available literatures, aiming to provide a compendium to clarify the possible benefits of targeting Th17/IL-17 to develop novel treatments for these patients (Fig. 1). A total of 146 reports were retrieved by the following keywords: “TH17”, “IL-17”, “Parkinson’s disease”, “PD”, “Alzheimer’s disease”, “AD”, “Amyotrophic lateral sclerosis”, “ALS”, “neurodegenerative diseases”. Finally, six studies [65–70] on targeted therapy for IL-17 were screened out (Table 1).

**Table 1:** Anti-IL-17 and IL-17R

Disease	Model	Intervention	Result	References
AD	LPS treated rat	IL‐17Ab	IL-17A Abs improved LPS-induced memory impairment	[64]
AD	Vivo animal model Inject Aβ1-42 into the ventricle	IL‐17Ab, IgG2A, clone 50104 Control: IgG2A, clone 54447	Preconditioning with IL-17Ab significantly reduced neurodegenerative changes induced by Aβ 1–42, improved memory function, and inhibited the increase of pro-inflammatory mediators in a dose-dependent manner Administration of IL 17Ab after a β 1–42 injection reduced neurodegenerative memory decline and pro-inflammatory mediators and cytokines levels	[65]
PD	HiPSC-derived neurons of PD	Anti-IL-17/antiIL-17R/secukinumab+ T lymphocyte Control: + T lymphocyte Blank control group	Anti-IL-17Ab, anti-IL-17R, and secukinumab reduced T lymphocyte-induced neuronal death, with no significant difference in cell death levels compared to neurons cultured without T cells	[66]
PD	MPTP-treated mice MPP+-treated rats	Anti-IL-17Ab	Injection of anti-IL-17Ab into the lateral ventricle of PD rats can improve the activation and dyskinesia of microglia in BBB-disrupting dopaminergic neurodegeneration	[67]
PD	MPTP-induced PD mice	Anti-IL-17Ab	Anti-IL-17Ab eliminated th17-induced death of DAergic neurons	[68]
ALS	iPSC-derived MNs (ALS)+ TH-17(ALS/HCs/MS)	Anti-IL-17Ab and anti-IL-17R	Th17 cells and IL-17A did directly promote MN degeneration. Anti-IL-17Ab and anti-IL-17R therapy reversed all effects of IL-17A	[69]

### Th17 cells and IL-17A in AD

AD is the most common neurodegenerative disease, contributing up to 70% of all cases of dementia, and has an exponentially increasing prevalence after the age of 65. Pathologically, AD is characterized by the deposition of extracellular senile plaques composed of amyloid-β (Aβ) and intracellular neurofibrillary tangles, resulting from the accumulation of hyperphosphorylated tau. Till now, there is no definite IL-17A alteration in AD patients. Some studies found that the IL-17A levels in the serum, brain and CSF of AD patients were increased, but other studies reported reduced IL-17A levels in AD patients. The conflicting results may be due to the lack of clinical dementia rating [71], but a recent meta-analysis showed a negative correlation between the disease progression of AD and the CSF IL-17A level [72]. Still, studies showed that plasma IL-17 levels could be used as a plasma biomarker to distinguish AD patients from cognitively healthy individuals [73], and CSF IL-17 concentrations could be used to identify frontotemporal lobar degeneration (FTD) with tau pathology [74]. Activated Th17 cells in the CNS could produce pathogenic cytokines IL-17A, recruit neutrophils, heighten the inflammatory cascade, and promote AD neuroinflammation and neurodegeneration [75, 76]. The genetic variations are considered to be an important candidate to induce AD via upregulation of IL-17A [77]. Increasing evidence showed that IL-17 did play a role in the neuronal degeneration of AD; the mechanisms included Aβ interaction, microglia activation, BBB disruption, systemic neuroinflammation, etc. [77].

#### Interaction with Aβ

The insoluble Aβ peptides could promote the production of reactive oxygen and nitrogen species, resulting in Th17 cells stimulation and IL-17 production [75, 78]. A previous study developed Aβ-reactive Th17 Teff cells, and adoptively transferred them into amyloid precursor protein/presenilin1 (APP/PS1) transgenic AD mice; the result showed that Aβ-specific Th17 Teffs cells play a role as disease perpetrators [79]. In the rat model generated by Aβ injection, IL-17 level was increased in both the circulation and CSF, and was also correlated with the cognitive function decline, indicating that Th17/Tregs balance was disrupted in AD [80]. Similarly, intraperitoneal injection of lipopolysaccharide (LPS) to transgenic mice (Tg2576), which overproduces human Aβ and develops plaques, resulted in the increase of eight cytokines, including IL-17A [81]. In an AD mouse model, Aβ aggregates were shown to mediate the recruitment of neutrophils in the CNS. The effects of IL-17A in AD pathogenesis are highly related to the attraction of neutrophils and the stimulation of neutrophils’ function [82]. Neutrophils were found to be present in areas with Aβ deposits, and this extravasation could lead to amplification of neutrophil entry in the CNS and IL-17 production [3]. In vitro study showed that IL-17A played a role in promoting neuronal autophagy and inducing neurodegeneration [83]. A previous study tested the therapeutic effect of salidroside (SAL), an herb-derived phenylpropanoid glycoside compound, in the senescence-accelerated mouse prone 8 (SAMP8) strain, which is a reliable and stable mouse model of AD strain, a reliable and stable mouse model of AD. The results showed that SAL decreased the IL-17A levels in the peripheral circulation, and alleviated hippocampus-dependent memory impairment [84].

#### Microglia activation

It was shown that exposure of microglia to IL-17A led to activation and increased production of pro-inflammatory cytokines [65]. In vitro study also showed that TLR-dependent activation of microglia could polarize γδ T cells toward neurotoxic IL-17+ γδT cells [85]. Periodontal bacteria were shown to be able to induce Aβ-42 accumulation and IL-17 expression in the cortex; IL-17 expression in microglia was negatively correlated with the memory test latency and positively correlated with Aβ-42 accumulation [86].

#### BBB disruption

IL-17A could disrupt BBB integrity by reducing BBB tight junctions (TJ) molecules and disrupting oxidant–antioxidant balance [61, 62]. The endothelial cells of BBB could express IL-17A receptors, and the binding of IL-17A to the receptors could cause the disruption of TJ [63] and downregulate the expression of the TJ molecules [62]. Blocking L-17A could reduce the BBB disruption and reverse the decrease of TJ molecules [64]. Zhang et al. constructed an AD rat model by intrathecal injection of Aβ-42 peptide, and they found that Th17 cells entered into the CNS with the disruption of the BBB, and levels of IL-17 and RORγt were increased in the hippocampus, CSF, and serum [87]. With the disruption of BBB integrity, more neutrophils and Th17 cells will migrate into the brain parenchyma, leading to more IL-17A production and more severe neuronal dysfunction [75, 78].

#### Systemic neuroinflammation

In a transgenic mouse model of AD, the activation of T and B lymphocytes was increased [88], and these lymphocytes could produce high levels of IL-2, granulocyte macrophage-colony stimulating factor (GM-CSF), tumor necrosis factor-alpha (TNF-α), and IL-17, which pointed to the Th17 polarization [88]. In AD patients, the number of CD4+ and CD8+ T lymphocytes was increased in vascular endothelium and brain parenchyma [89]. The production of Th17-related cytokine IL-21 was increased and the expression of Th17 transcription factor RORγt was upregulated in naive lymphocytes obtained from AD patients [90]. In AD patients, the circulating CD3+CD8−IL-17A+interferon-gamma (IFN-γ)−Th17 cells were found to be increased, indicating that the adaptive immune system is related to neuropathological changes in AD [91]. In an animal model, LPS injection in male Sprague-Dawley (SD) rats increased the IL-17A expression in serum and in the hippocampus [65]. It was reported that inadequate immune surveillance in the gut [92] or respiratory infection [93] could induce higher IL-17A production in the CNS, which would result in Aβ deposition. However, there may be opposite sequences of events, Aβ deposition and inadequate clearance of Aβ would stimulate the receptors of innate immune cells, promote IL-17 production, and induce AD pathogenesis. In a double transgenic APΔE9 mouse with overproduced Aβ, a higher frequency of CD4+ IL-17a and IFN-γ secreting T cells was revealed in the brain, indicating T-cell infiltration may be associated with the neuroinflammatory state in AD [94]. In AD patients, cognitive impairment progression was found to be related to Th17 cells and c-Jun N-terminal kinase (JNK) pathway-associated phosphatase (JKAP), the latter play a key role in regulating inflammation and immune responses; JKAP and Th17 cells were dysregulated and inter-correlated in AD [95]. Some compounds from plants could reduce the associated neuroinflammation in AD. For example, acid alpha-glucosidase (GAA), a kind of lanostane-type triterpenoid isolated from Ganoderma lucidum, was found to have an alleviating neuroinflammatory effect on AD mice via regulating the imbalance of the Th17/Tregs axis [96]. OMT, an alkaloid component extracted from the root of Sophora flavescens Ait, could reduce the level of pro-inflammatory cytokines including IL-6, IL-1β, TNF-α and IL-17A in AD mice [97]. Kavalactones, extracted from the rhizome and roots of kava, could decreased microgliosis, astrogliosis and secretion of the pro-inflammatory cytokines TNF-α and IL-17A, and attenuated the long-term memory decline of APP/Psen1 mice [98].

#### Strategic therapy targeting on IL-17A

In an AD mouse model, administration of anti-IL-17A antibody to block IL-17A generation could decrease the neuroinflammation induced by Aβ-42 injection, reduced neuronal neurodegeneration, and improve the cognitive impairment of the mice [66]. In male SD rats injected intraperitoneally with LPS, the use of IL-17A-neutralizing antibodies inhibited the expression of APP and Beta-site APP-cleaving enzyme 1 (BACE1), and prevented the expression of TNF-α, IL-6 and inflammatory proteins, indicating the role of anti-IL-17A strategy in the treatment of endotoxemia-induced neuroinflammation and cognitive dysfunction [65]. Anti-IL-17A antibodies could also interfere with neutrophil infiltration into the CNS and inhibit AD progression [99]. It was proposed that a desirable AD vaccine should be effective in inhibiting Th17/IL-17A immune responses to Aβ deposition aiming to limit the neuroinflammation in neurodegeneration [100]. A systemic review reported that none of the current AD drugs is specifically designed to target the dysregulated balance in the Th17/Treg axis, indicating that future therapeutic approaches should specifically consider inhibiting CD4+ Th17 in AD [101]. However, a protective role of IL-17A was also indicated in an animal model of AD, and overexpression of IL-17A intracranially could reduce cerebral amyloid angiopathy, improve glucose metabolism, decrease soluble Aβ levels in the hippocampus and CSF, relieve anxiety, and improve learning deficits [102]. Further, injected ICR mice with IL-17 could improve spatial learning, indicating a complex role of IL-17 in regulating adult neurogenesis [103]. These above-mentioned findings indicate that the role of IL-17A in AD is complicated, it may switch from a protective role to a pathogenic role depending on the disease state.

### Th17 cells and IL-17A in PD

PD is the second most common neurodegenerative disease after AD, characterized by the progressive degeneration of dopaminergic (DA) neurons within the substantia nigra pars compacta (SNpc) in the midbrain, the formation of Lewy bodies with aggregated a-synuclein in intracellular inclusions, and the presence of neuroinflammation [104–109].

In PD, Th17 cells were assessed by means of surface markers or intracellular IL-17 staining. The former found similar or even reduced Th17 cells in PD patients [110–112], while the latter reported increased Th17 cells in PD patients or no differences in Th17 cells between PD patients and healthy subjects [67, 113–116]. Therefore, the results of different studies concerning the frequency of Th17 between PD patients and controls were contradictory, but the published studies consistently reported an increased frequency of IL-17-producing cells in PD patients [117]. However, some studies reported decreased plasma levels of the IL-17A in PD patients [118, 119].

#### Interaction with a-synuclein

In a mitochondrial permeability transition pore (MPTP) mouse model of PD, stimulation of Th17 cells with a-synuclein could cause neuronal cell death in the substantia nigra (SN) [119]. Liu et al. used rabies virus glycoprotein (RVG) peptide-modified exosome (EXO) curcumin/phenylboronic acid-poly(2-(dimethylamino)ethyl acrylate) nanoparticle/small interfering RNA targeting SNCA (REXO-C/ANP/S), a nano-scavenger for clearing α-synuclein aggregates in neurons, as a platform for PD treatment, and they found that REXO-C/ANP/S could achieve immune activation clearing by inhibiting Th17 and enhancing Treg to regulate the immune system in mice with PD [120].

#### Involvement of peripheral immune cells

The involvement of peripheral immune cells [121] was reported in PD patients [122]. A previous study found that global or CD4+ T cell-specific dopamine 2 receptor (DRD2) deficiency could exacerbate MPTP-induced dopaminergic neurodegeneration and CD4+ T-cell depolarization towards pro-inflammatory Th17 phenotypes, indicating that DRD2 expressed on CD4+ T cells is protective against neuroinflammation and developing a therapeutic strategy of stimulating DRD2 may be promising for amelioration of Th17-inflammatory response in PD [123]. In vitro analyses showed about threefold increase in Th17 cells frequency, a phenotype favored by DRD3-signalling, in ex vivo activated CD4+ T cells obtained from PD patients, indicating that DRD3-signalling in lymphocytes plays a relevant role favoring the development of PD, and selective DRD3-antagonism in CD4+ T lymphocytes may exert a therapeutic effect in PD [124]. Vitamin D was reported to inhibit the production of IL-17 and IFN-γ, and promote the differentiation and function of Treg in both rodent and human T cells [125–127]. The vitamin D-induced benefits in PD might partly depend on its immune effects of Th17 and Treg cells [117], but more studies are needed to verify the exact contribution of vitamin D in modulating Th17 and Treg in PD.

#### Neurotoxic effects

In cell models of PD, the co-culture of Th17 cells with MPTP-treated neurons could exacerbate neuronal cell death [68, 128]. Sommer et al. had shown that Th17 cells obtained from PD patients induced neuronal death in the midbrain, indicating the neurotoxic effect of Th17 cells in PD [67], and this neurotoxicity of Th17 cells was driven by T cell-derived IL-17, upregulated IL-17R, downstream Nuclear factor-kappa-B (NFκB) activation, as well as lymphocyte function-associated antigen-1/intercellular adhesion molecule-1(LFA-1/ICAM-1) system [67, 119]. Moreover, the rescue of Th17-mediated neuronal death could be achieved by blocking ICAM-1 and IL-17R, or by blocking LFA-1 and IL-17 in Th17 cells with anti-IL-17 antibodies, directs us toward new potential immunotherapeutic targets for PD [67, 119].

#### BBB disruption

In PD patients, the disruption of BBB was reported [122], and the increased permeabilization of BBB allowed infiltration of peripheral immune cells into the CNS [129]. In PD animal models, BBB was disrupted and IL-17A level was increased in the SN [68].

#### Systemic neuroinflammation

Previous studies had found increased circulating Th17 cells in PD patients at the early stages of the disease [84, 104], indicating an important role of Th17-driven inflammation in PD. Furthermore, in Porphyromonas gingivalis (Pg)-treated leucine-rich repeat kinase 2 (LRRK2) R1441G mice, dopaminergic neurons in the SN were reduced, but serum IL-17A, brain IL-17 receptor A, and activated microglial cells were increased; these findings indicated that neuroinflammation might play an important role in the pathophysiology of LRRK2-associated PD [130]. A previous study showed that auricular vagus nerve stimulation (aVNS) treatment decreased Th17 cells, and reduced the levels of inflammatory cytokines, including TNF-α and IL-1β in 6-OHDA treats rats, indicating that aVNS could suppress the evolution of inflammation and modulating innate immune responses to play a neuroprotective role against dopaminergic damage [131]. In PD mice, administration of purified bee venom (BV) phospholipase A2 (bvPLA2) inhibited loss of dopaminergic neurons within the SN in a dose-dependent manner, and this concentration-dependent action appeared to be related to the inhibition Th17 polarization; these results suggest that standardized bvPLA2 may have a neuroprotective effect against PD through neuroinflammation modulation [132]. JKAP, the regulator of immunity and inflammation, was also found to be correlated with Th17 cells and disease severity in PD [133]. Repetitive transcranial magnetic stimulation (rTMS) was proved to have therapeutic effects on neuroinflammation via reducing the production of pro-inflammatory cytokines IFNγ and IL-17A [134].

#### Microglia activation

Addition of IL-17A to co-cultures of microglia and neurons led to activation of microglia cells and TH+ neuronal cell death. Interestingly, IL-17A exacerbated dopaminergic neuronal loss only in the presence of microglia. Furthermore, the inhibition of the IL-17A receptor on microglia was sufficient to attenuate these effects [68]. A network of communication may exist between glial cells and Th17 cells, a greater understanding of this interaction may provide a novel therapeutic approach [135]. A previous study found that High mobility group protein B1 (HMGB1) A box inhibited the activation of microglia-mediated by HMGB1, inhibited the infiltration of Th17 cells, and decreased the proportion of Th17 in CD4+ T cells, indicating that HMGB1 A box may play a different role in protecting neurons in PD via influencing the activation of microglia cells, the infiltration of Th17 cells, and the differentiation of T cells to Th17 [136].

#### Alteration of gut microbiota

Altered gut microbiota was described in PD patients, and it also had strong potential to mediate motor deficits and neuroinflammation in PD model [137]. Furthermore, intestinal microbiota has the ability to induce Th17 differentiation [37]. Therefore, the specific Th17 cells and their role in directing against gut microbiota might inspire the development of gut immunomodulatory therapeutic approaches in PD patients [129].

#### Strategic therapy targeting on IL-17A

In the experimental phase, the anti-Th17 therapeutics in PD can be achieved by using nuclear receptor agonists, including peroxisome proliferator-activated receptor gamma (PPARγ) and liver X receptor (LXR), both of them are known to negatively regulate differentiation of Th17 cells [138, 139]. These agonists may have therapeutic prospects in PD because they effectively inhibit PD pathology [140]. Furthermore, an anti-IL-17A-neutralizing antibody proved to be effective in alleviating the PD manifestations in the PD rat model [68].

### Th17 cells and IL-17A in ALS

ALS is a neurodegenerative disorder characterized by progressive degeneration of upper and lower motor neurons (MNs), resulting in muscle weakness and paralysis. The possible involvement of Th17 in ALS is indicated by circumstantial evidence. Studies had shown increased IL-17 levels in serum and CSF of ALS patients, and in the cell model, the IL-17 production was upregulated by cultured peripheral blood mononuclear cells [141, 142]. ALS patients had a higher expression of IL-17A in serum than controls, indicating a greater vulnerability of ALS patients to IL-17A-mediated damage. In ALS patients, the immune profile in peripheral blood was shifted towards a Th1/Th17 cell-mediated pro-inflammatory immune response, and Th1 and Th17 cells were moderate negatively correlated with disease severity, evaluated by forced vital capacity and ALS functional rating scale revised (ALSFRS-R) [143]. The spinal cords of ALS patients were found to be infiltrated by IL-17A-positive CD8 cells and IL-17A-positive mast cells. Mononuclear cells treated with aggregated superoxide dismutase-1 (SOD-1) protein could induce the expression of IL-6, IL-23, and IL-1β, which may be responsible for the induction of IL-17A [144]. IL-17A may be involved in chronic inflammation in ALS, and could be a new therapeutic approach by immune modulation of inflammatory cytokines.

#### Strategic therapy targeting on IL-17A

A recent study developed a co-culture system of human-induced pluripotent stem cells (hiPSCs)-derived MNs and Th17 cells, derived from ALS patients, MS patients, and healthy controls. They found that Th17 cells from MS patients induced severe degeneration of MNs, and IL-17A yielded a decline of viability and neurite length of MNs in a dose-dependent manner. Furthermore, neutralizing IL-17A and anti-IL-17A receptor treatment reverted this detrimental effect of IL-17A [143].

## Conclusions

In 2021, we compared 761 age–gender matched healthy controls with 761 PD patients and found that the ratio of CD4/CD8 in PD patients was higher than that in healthy controls, and the percentage of CD4+ T cells was negatively correlated with the Hoehn and Yahr (H&Y) stage [145]. However, we did not compare the subtypes of CD4+ T cells. Although the function of TH17/IL-17A on AD or PD is still contradictory and the mechanism of TH17/IL-17A is still unclear, the results of the latest research on IL-17A targeted treatments are still valid, so the pathogenesis and targeted therapy of IL-17A in neurodegenerative diseases are still worth exploring.

## Data Availability

No.

## Abbreviations

AD: Alzheimer’s disease
PD: Parkinson’s disease
ALS: Amyotrophic lateral sclerosis
CNS: Central nervous system
TH17: T helper 17
IL-17A: Interleukin-17A
TGF-β: Transforming growth factor-β
ROR: Retinoic orphan receptor
CTLA: Cytotoxic T-lymphocyte antigen
TCR: T-cell receptor
NKT: Natural killer T
ILC3: Group 3 innate lymphoid cells
CXCL: C-X-C motif ligand
G-CSF: Granulocyte colony-stimulating factor
AS: Ankylosing spondylitis
RA: Rheumatoid arthritis
SLE: Systemic lupus erythematosus
IBD: Inflammatory bowel disease
MS: Multiple sclerosis
EAE: Experimental autoimmune encephalomyelitis
CSF: The cerebral fluid
BBB: The blood–brain barrier
Aβ: Amyloid-β
FTD: Frontotemporal lobar degeneration
APP/PS1: Amyloid precursor protein/presenilin1
LPS: Lipopolysaccharide
Tg: Transgenic
SAL: Salidroside
SAMP8: Senescence-accelerated mouse prone 8
TJ: Tight junctions
GM-CSF: Granulocyte macrophage-colony stimulating factor
TNF-α: Tumor necrosis factor-alpha
IFN-γ: Interferon-gamma
SD: Sprague-Dawley
JNK: C-Jun N-terminal kinase
JKAP: C-Jun N-terminal kinase (JNK) pathway-associated phosphatase
GAA: Acid alpha-glucosidase
BACE1: Beta-site APP-cleaving enzyme 1
DA: Dopaminergic
SNpc: Substantia nigra pars compacta
MPTP: Mitochondrial permeability transition pore
SN: Substantia nigra
REXO-C/ANP/S: Rabies virus glycoprotein (RVG) peptide-modified exosome (EXO) curcumin/phenylboronic acid-poly(2-(dimethylamino)ethyl acrylate) nanoparticle/small interfering RNA targeting SNCA
DRD2: Dopamine 2 receptor
NFκB: Nuclear factor-kappa-B
LFA-1/ICAM-1: Lymphocyte function-associated antigen-1/intercellular adhesion molecule-1
Pg: *Porphyromonas gingivalis*
LRRK2: Leucine-rich repeat kinase 2
aVNS: Auricular vagus nerve stimulation
bvPLA2: Bee venom (BV) phospholipase A2
rTMS: Repetitive transcranial magnetic stimulation
HMGB1: High mobility group protein B1
PPARγ: Peroxisome proliferator-activated receptor gamma
LXR: Liver X receptor
MNs: Motor neurons
ALSFRS-R: Amyotrophic lateral sclerosis functional rating scale revised
SOD-1: Superoxide dismutase-1
hiPSCs: Human-induced pluripotent stem cells
H&Y: The Hoehn and Yahr
